# Preparation and Evaluation of Pullulan/*Astragalus* Extracts Bioactive Food Packaging Incorporated with β-Cyclodextrin and Its Anti-Browning in Apples

**DOI:** 10.3390/foods15132373

**Published:** 2026-07-03

**Authors:** Shuyan Zhang, Shihao Chen, Yingyin Wu, Wangying Yan, Yulian Ye, Jie Zhu, Shilin Liu

**Affiliations:** 1 College of Food Science & Technology, Huazhong Agricultural University, Wuhan 430070, China; zhangsy@dgut.edu.cn; 2 Dongguan Key Laboratory of Typical Food Precision Design, China National Light Industry Key Laboratory of Healthy Food Development and Nutrition Regulation, School of Life and Health Technology, Dongguan University of Technology, Dongguan 523808, China; spcsh2020@163.com (S.C.); z511wyy@163.com (Y.W.); 15989306380@163.com (W.Y.); 19507654703@163.com (Y.Y.)

**Keywords:** β-cyclodextrins, *Astragalus* extract, anti-browning, food packaging

## Abstract

Polysaccharides-based bioactive films are regarded as a promising strategy to delay fruit spoilage. β-cyclodextrins (CD) was incorporated into pullulan/*Astragalus* extract (AE) bioactive films (CD-AE-P) to systematically investigate its effect on multi-scale structures features, physicochemical properties, migration behavior, anti-browning efficacy, and aroma substances in sliced apples. With increasing CD content, a slight blue shift was observed in O-H stretching vibration peak; CD-AE-P films retained an amorphous structure, as evidenced by the disappearance of scattering peak at 0.075 < *q* < 0.25 nm^−1^, and they exhibited smoother, continuous fractured surfaces with sparsely distributed protrusions. Meanwhile, CD incorporation contributed to the preservation of macromolecular thermal stability and induced an initial increase followed by a decrease in storage modulus (*E*′), with CD15-AE-P sample exhibiting the highest Tg value. Although the hydrophilicity increased marginally in the films upon adding CD, WVP decreased progressively, reaching a 23.3% reduction in CD25-AE-P relative to CD0-AE-P. Furthermore, total migration from the films was significantly suppressed by CD incorporation, and the anti-browning effect on sliced apples was extended; notably, treatment with CD25-AE-P effectively preserved the aroma substances of sliced apples during storage. These results demonstrate that CD serves as an effective functional carrier to modulate the effective release of bioactive compounds in active food packaging systems.

## 1. Introduction

Postharvest deterioration, including microbial spoilage, enzymatic browning, and progressive nutrient loss, compromises key quality attributes of the flavor, notably color, texture, and microbiological safety in fruits [1,2]. Critically, surface browning (or blackening) is widely recognized as a primary visual indicator of quality decline and a decisive factor governing consumer acceptance and marketability [3,4].

Polymer-based bioactive and intelligent packaging technologies have emerged as highly promising preservation platforms, driven by escalating consumer demands for safer, higher-quality, and more sustainable fresh produce [5,6]. Which showed benefits in controlled delivery of natural antimicrobials and antioxidants, modulating key postharvest physiological processes: moisture migration, gas permeability (O_2_/CO_2_ exchange), enzymatic browning reactions, and microbial proliferation [7,8,9]. Such multifunctional regulation is beneficial to suppressing surface discoloration and preserving nutritional integrity, making biopolymer-based bioactive packaging a focal area of innovation in fruit and vegetable preservation [10,11].

Bioactive compounds are the substances found in foods or plant-derived substances with documented functional properties, as they confer multiple advantages in food packaging, including extended shelf life, enhanced food safety, effective inhibition of enzymatic browning, and preservation of nutritional quality [8,12]. *Astragalus*, a perennial leguminous herb native to East Asia, is abundant in bioactive constituents, including flavonoids, astragalosidic saponins (AS), and polysaccharides (ASP), and it has been traditionally revered in Chinese medicine for over two millennia as a tonic herb with adaptogenic and immunomodulatory properties [13,14]. In the meantime, it has received approval for use as a food ingredient by both the National Health Commission of China and the European Food Safety Authority (EFSA) Panel on Nutrition [15,16]. In food packaging research, *Astragalus* gum has been shown to enhance the water solubility, water vapor permeability, tensile strength, and structural homogeneity of polyvinyl alcohol (PVA)-based nanocomposite films [17,18]. Additionally, *Astragalus* saponin, a key bioactive component of *Astragalus* extract (AE), is increasing incorporated into polysaccharide-based films owing to their distinctive molecular structure and well-documented antioxidant and antibacterial activities [19,20]. As the most abundant and functionally versatile class of bioactives in AE, *Astragalus* polysaccharides (ASP) exhibit a broad spectrum of physiological activities, including immunomodulation, hypoglycemic, anti-inflammatory, antioxidant, and antiviral effects, making them a compelling subject of current investigation in active packaging and nutraceutical delivery [21,22]. Electro-spun nanofibers composed of polyvinyl alcohol (PVA), *Astragalus* polysaccharide (ASP), and astragaloside IV (AS) have been demonstrated to enhance the deposition of collagen fibers, facilitate the repair of regenerated epithelium, and promote wound healing in diabetic rats [23]. Therefore, active packaging incorporating AE holds promising potential for extending the shelf life of fruits within polysaccharide-based food packaging system. However, the inherent incompatibility between hydrophobic AS and hydrophilic ASP poses a significant formulation challenge, limiting the homogeneous dispersion and functional efficacy of AE and thereby constraining its broader practical application. β-cyclodextrins (CD) are cyclic oligosaccharides with a truncated cone-shaped structure, featuring a hydrophilic external surface and a relatively hydrophobic internal cavity [24]. The hydrophobic internal cavity of β-cyclodextrins enables efficient encapsulation of lipophilic bioactive compounds, including natural antioxidant extracts and essentials oils (EOs), while their well-documented low toxicity and regulatory approval for food use support their safe incorporation into active food packaging systems [25,26]. Consequently, integrating CD with polysaccharide-based matrices containing AE offers a rational strategy to formulate bioactive packaging materials that simultaneously ensure homogeneous dispersion, sustained antioxidant activity, and effective preservation of perishable fruits.

Currently, AE was obtained via cellulase-assisted microwave extraction, the multi-scale structures, as well as physicochemical properties of pullulan/AE bioactive films with varying concentrations of β-cyclodextrins (CD-AE-P), were comprehensively characterized. Moreover, the migration behavior of CD-AE-P films was further assessed, and their anti-browning effect in fresh-cut apple slices and further changes of volatile organic compounds (VOCs) were validated by GC-IMS. These results broaden the functional application potential of AE in active food packaging and provide a practical strategy for extending the shelf life of postharvest fruits and vegetables.

## 2. Materials and Methods

### 2.1. Materials

*Astragalus*, was purchased from Beijing Tongren Hospital Health Pharmaceutical Co., Ltd. (Ningxia, China); Pullulan (P121048, weight-average molecular weight of approximately 200 kDa, with a typcial molecular weight distribution ranging from 48 kDa to 2200 kDa), sodium phosphate monobasic (NaH_2_PO_4_), astragaloside IV, protease, pectinase, glycerol, vanillin, Shanghai Aladdin Biochemical Technology Co., Ltd. (Shanghai, China); Citric acid (C_6_H_8_O_7_), β-Cyclodextrin (Mw = 1134.98), Shanghai yuanye Bio-technology Co., Ltd. (Shanghai, China); Concentrated sulfuric acid, Tianjin Damao Chemical Reagent Factory (Tianjin, China).

### 2.2. Extraction of Active Components from Astragalus

The extraction of active components from *Astragalus* was conducted using a combination of mixed enzymatic hydrolysis and microwave-assisted extraction. Firstly, 50.00 g of dried *Astragalus*, which have been oven-dried for 24 h, was grounded using a pulverizer for 2 min and subsequently sieved by an 80-mesh sieve to obtain homogeneous *Astragalus* powder. The *Astragalus* powder (2.0 g) was then transferred into a beaker containing 60 mL of 70% ethanol solution to prepare the extraction mixture. Secondly, the pH of the mixture was adjusted to 5.2 using a disodium hydrogen phosphate–citric acid buffer solution. A defined quantity of mixed enzyme solution (cellulase:pectinase:protease = 1:2:1, 10%, *w*/*w*, based on *Astragalus*) was added to the solution, and enzymatic hydrolysis was performed at 52 °C for a duration of 2 h. Thirdly, the enzymatically treated mixture underwent microwave irradiation at varying power levels (400 W, 500 W, 600 W, 700 W) for periods of either 80 s, 100 s, and 120 s, respectively. Finally, the filtered supernatant-containing bioactive compounds (Astragalosides, AST; *Astragalus* polysaccharides, APS) were freeze-dried to yield samples of *Astragalus* extract (AE), which were subsequently stored in sealed containers.

### 2.3. Preparation of Pullulan-AE Based Bioactive Films Combined with β-Cyclodextrin

Firstly, 0.10 g, 0.15 g, 0.20 g, 0.25 g, and 0.30 g of β-cyclodextrin (CD) were placed into a beaker and magnetically stirred with 10 mL of deionized water for 60 min to ensure complete dissolution (600 r/min, 60 °C). Subsequently, 1.00 g of pullulan was added to the beaker and mixed thoroughly. After that, 0.6 g of AE and 0.2 g of glycerol were incorporated into the mixture and stirred for an additional 30 min (600 r/min, 30 °C) to achieve full dispersion. The film-forming solutions were then poured onto low-density polyethylene culture dishes (9 × 9 cm^2^), where small bubbles present in the mixtures were carefully punctured using needles. The solutions were dried in an oven set at 50 °C oven for 24 h. Thereafter, the dried films were equilibrated for 24 h (25 °C, RH = 50%) and stored in a desiccator for future use. The prepared pullulan–AE bioactive films with varying concentrations of CD were named as: CD0-AE-P, CD10-AE-P, CD15-AE-P, CD20-AE-P, CD25-AE-P, and CD30-AE-P.

### 2.4. Properties of AE

Extraction efficiency: The AST content was evaluated to determine the extraction efficiency of AE. AST exhibited the maximum absorption peak at a wavelength of 540 nm [27]. Precisely, 0.1 mL, 0.2 mL, 0.3 mL, 0.4 mL, and 0.5 mL of AST standard solutions with a mass concentration of 0.50 mg/mL were placed into tubes with stoppers. Ethanol was then added to adjust the total volume to 0.5 mL; subsequently, 0.5 mL of 8% vanillin solution and 5.0 mL of 72% sulfuric acid were then added and thoroughly mixed. The mixtures were incubated in a water bath at 62 °C for 20 min before being cooled to room temperature. The absorbance of the AST standard solutions was measured using a visible light–ultraviolet spectrophotometer (EVOLUTION 220, Thermo Fisher, Waltham, MA, USA). Following this procedure, an aliquot of 0.2 mL from the filtered solution prepared in Section 2.1 underwent identical treatment as that applied to the AST standard solutions, thus enabling determination of the AST content in AE through reference to the established standard curve.

Antioxidant capacity: The measurement was conducted following the research method established by Cui et al. [21] with minor modifications. Initially, 2 mL of 0.2 mmol/L DPPH solution were mixed thoroughly with 95% ethanol solution. Subsequently, 2 mL of AE were combined with 2 mL of 0.2 mmol/L DPPH solution and mixed adequately. Additionally, a mixture of 2 mL of AE and 2 mL of 95% ethanol solution was prepared and shaken well. The three resulting mixtures were then placed in darkness for a duration of 30 min, after which their absorbance values at 517 nm were recorded as *A*_0_, *A*_i_, and *A*_j_, respectively. The results were expressed as the DPPH radical scavenging rate (SR).

SR (%) = (1 − (*A*_i_ − *A*_j_)/*A*_0_) × 100%

### 2.5. Structural Characterization

FTIR: The FTIR spectra of CD-AE-P with varying CD content were analyzed using attenuated total reflectance (ATR). All films were measured with a Nicolet IS50 scientific instrument (Thermo Fisher, USA) over the range of 4000 to 400 cm^−1^, employing a resolution of 4 cm^−1^ and averaging 32 scans at room temperature.

XRD: CD-AE-P bioactive film samples were cut into square pieces with a size of 1 cm^2^. These samples were then flattened and positioned in the sample chamber of an X-ray diffractometer (MiniFlex 600, Rigaku, Akishima-shi, Japan), using Cu-Kα as the radiation source (λ = 0.1542 nm). The testing conditions included a voltage of 40 kV and a current of 30 mA. The scanning was conducted over a range from 5 to 50° with a step size of 0.02° and a scanning speed set at 4 °/min.

SAXS: The experiment was performed using an SAXSess small-angle X-ray scattering instrument (SAXS-NanoSTAR, Bruker, Karlsruhe, Germany). All CD-AE-P bioactive film samples were prepared in dimensions of 5 × 5 mm^2^ and placed in the SAXS sample holder slot equipped with a two-dimensional detector. Cu-Kα served as the light source (λ = 0.1542 nm), while the operating current and voltage were maintained at values of 40 mA and 45 kV, respectively; the testing duration was set for 120 s. Software was employed to convert the two-dimensional integral signal into one-dimensional function I(*q*) concerning the modulus $\vec{q}$ of the scattering vector, thereby facilitating acquisition of the I(*q*)~*q* scattering spectrum (with respect to *θ* as the scattering angle). Herein, *q* is defined as follows:

$$q=\;\left|\vec{q}\right|\;=\;(4\pi \sin\theta )/\lambda$$

SEM: The cross-sectional morphologies of CD-AE-P film samples were examined using a scanning electron microscope (EM-30 Plus, COXEM, Daejeon, Republic of Korea) operating at a voltage of 5 kV. Film samples were cut into square pieces measuring 0.25 cm by marking the edges. Subsequently, these marked samples were immersed in liquid nitrogen to achieve natural fracture. The fractured specimens were then positioned upright and coated with gold via sputtering for 1 min to avert charging prior to the test. The magnification was set and maintained at 1000×.

### 2.6. Physico-Chemical Properties

Thermal Stability: The thermal gravimetric analysis (TGA 8000, PE, Shelton, CT, USA) was employed to assess the thermal properties of CD-AE-P film samples. The heating range was set from 30 °C to 600 °C with a heating rate of 10 °C/min. Nitrogen was selected as the purge gas at a flow rate of 25 mL/min. Each sample underwent three tests to obtain an average value.

Dynamic Mechanical Analysis: Appropriately sized strips of film samples were measured for thickness and width before being placed in the sample chamber of DMA (DMA8000, PE, USA) instrument. The temperature ranged from −30 °C to 100 °C with a heating rate of 5 °C/min, the frequency was maintained at 1 Hz with a strain of 0.02 mm, and the static force and dynamic force applied were set at 0.05 N and 2.00 N, respectively, while the drive control mode operated under automatic tension conditions. Results recorded included storage modulus (*E′*), loss modulus (*E″*), and loss factor (Tanδ).

Water Vapor Permeability: Water vapor permeability of the films was determined using the ASTM E96/E96M-24 method (i.e. Gravimetric Determination of Water Vapor Transmission Rate of Materials, American Society of Testing and Materials, West Conshohocken, PA, USA, 2024.3) with minor modifications. Initially, 2 g of anhydrous CaCl_2_ (0% RH) were added to glass cups (dimensions: inner diameter = 25 mm, depth = 55 mm), which were then sealed on the top and weighed (*m*_1_). These glass containers were subsequently placed in a closed environment filled with saturated potassium sulfate solution. The desiccator was then incubated at a constant temperature of 25 °C to maintain stable water vapor pressure Δp (approximately 3168 Pa). After 16 h, the weight (*m*_2_) of each glass container was measured.

WVP = Δm × *d*/(A × *t* × Δp)

Thereinto, Δ*m* represents the weight change (g) before and after the testing, *A* is the area of the glass bottle mouth (cm^2^), *t* is the testing time (s), *d* is the testing thickness (mm), and Δp was the partial pressure difference (kPa) across both sides of the film.

Water Contact Angle: The WCA was measured using a contact angle meter (PWEREACH, Zhongchen Digital Technology Co., Ltd., Shanghai, China). CD-AE-P film samples (20 × 20 mm^2^) were carefully adhered to a movable horizontal platform of the contact angle meter. Deionized water was dripped onto the surface of the samples with a 5 μL micro-injector, and the image was captured at the moment when the droplet fell on the film surface.

### 2.7. Migration Behavior

Overall migration: The test of EU Regulation No 10/2011 was used to analyze the gravimetric overall migration [28]. Considering the hydrophilic property of CD-AE-P film samples, the samples (1 × 1 cm^2^) were immersed in 10 mL of food simulations (distilled water) in closed glass containers. In the meantime, the samples were immersed in distilled water for 1 h. After the test period, 10 mL of the simulated solutions were transferred into pre-weighted glass tubes and evaporated at 100 °C, with a nitrogen flush using a heat-evaporator. The final weight of the glass tubes was then measured, and the overall migration values were calculated by %. All samples were tested in triplicate.

### 2.8. Application of Fresh-Cut Apple Preservation

Fresh apples were sliced and divided into three groups: the blank group immersed by distilled water (CK1), the pullulan film-forming solution (CK2), and the CD-AE-P film-forming solution. All treated apple slices were placed at room temperature to accelerate browning; the surfaces of pre-treated apple slices were pictured every 2 h to evaluate the anti-browning effect by CD-AE-P.

### 2.9. Fingerprint Analysis

The headspace-gas chromatography-ion mobility spectrometry (HS-GC-IMS) method was employed to determine the volatile compounds of sliced apples stored at 4 °C for 48 h. Briefly, 2 g of processed apple slices were placed in a headspace container (20 mL) and incubated at 60 °C for 15 min. The measuring parameter (analysis time, the type of column, IMS temperature, injection volume/needle temperature) and GC analysis parameters were corresponding with Zhang et al.’s formulation [29]. In addition, a two-dimensional cross-qualitative method was employed to identify the volatile compounds by comparing their RI and drift time to the GC-IMS library (NIST and IMS databases).

### 2.10. Statistical Analysis

All data were processed using IBM SPSS Statistics Version 20.0 and presented as mean ± standard error. Statistical analysis was performed using one-way analysis of variance (ANOVA), followed by Duncan’s post hoc test to determine differences between means. A *p*-value < 0.05 was considered statistically significant.

## 3. Results and Discussion

### 3.1. Extraction Efficiency and Oxidation Resistance of AE

**Figure 1:**
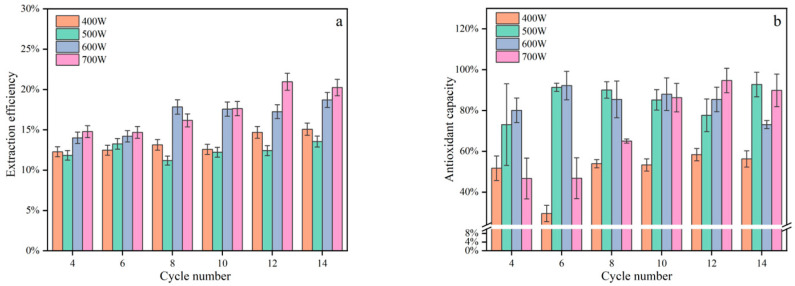
The extraction efficiency (**a**) and antioxidization capacity (**b**) of AE prepared by enzymatic hydrolysis combined with microwave treatment.

A calibration curve for astragaloside quantification was established as at 540 nm (y = 3.3217*x* − 0.0006, *R*^2^ = 0.9949). This linear relationship was applied to determine the astragaloside content in AE obtained via enzymatic digestion coupled with microwave-assisted extraction, which was conducted at four power levels (400 W, 500 W, 600 W, and 700 W) and six irradiation cycle numbers (4, 6, 8, 10, 12, and 14), with each cycle lasting 30 s (Figure 1a). At any fixed microwave power, the extraction yield of astragaloside was observed to increase progressively with extended irradiation time, reaching the highest accumulation of 20.96% at 700 W. Conversely, at a given cycle number, the extraction efficiency was consistently enhanced by higher microwave power, and the most pronounced improvement was observed after 12 cycles across all power levels. Overall, the maximum astragaloside yield of 20.96% was achieved under the optimized combined conditions of 700 W and 12 irradiation cycles.

Figure 1b presented the DPPH radical scavenging activity of AE obtained via microwave-assisted extraction, which was conducted across a range of microwave power levels and irradiation cycle numbers. At 400 W, the DPPH scavenging activity remained consistently low and nearly invariant (50%) across all cycle numbers, indicating limited antioxidant activation under low-power conditions. In contrast, markedly higher activities were observed at intermediate powers: peak values of 97.05% (achieved at 500 W and 14 cycles) and 96.33% (achieved at 600 W and 6 cycles). Furthermore, a distinct power-dependent trend was observed at 700 W, where the scavenging activity exhibited a significant positive correlation with irradiation time, reaching a maximum of 92.67% after 12 cycles. Crucially, when simultaneous optimization for both astragaloside yield and DPPH scavenging activity, the extraction condition of 700 W for 12 cycles was identified as the optimal-effectively balancing high recovery of bioactive compounds with superior antioxidant functionality.

### 3.2. Multi-Scale Structures of CD-AE-P Films

#### 3.2.1. Ftir Analysis

**Figure 2:**
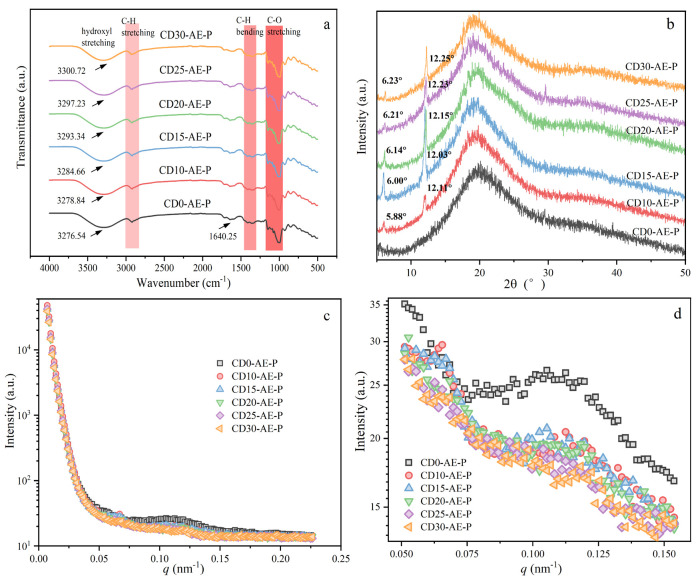
FTIR (**a**), XRD (**b**), and SAXS (**c**) spectrum of AE-P bioactive films with varying concentrations of CD; Partial enlarged drawing of SAXS spectrum (**d**).

ATR-FTIR spectroscopy is a widely employed analytical technique for probing molecular-level structural features of polymer blends [30,31]. Characteristic FTIR absorption bands of β-cyclodextrins (CD) include: C-H stretching vibration at 2926 cm^−1^, asymmetric and symmetric O-H stretching vibration in the range of 3300–3400 cm^−1^, and symmetric C-O-C stretching vibration at 1023 cm^−1^ [32]. Figure 2a displayed the FTIR spectra of pullulan/AE-based bioactive films incorporating varying concentrations of CD. All spectra exhibited canonical polysaccharide signatures: a broad band centered at 3200–2600 cm^−1^ was assigned to overlapped O-H stretching vibration arising from intramolecular hydrogen bonding and residual moisture; a distinct peak at 2919 cm^−1^ featured C-H stretching, highlighted in light red, and a sharp band at 1645 cm^−1^ featured H-O-H bending vibration of physically absorbed water [29]. Additional spectral features include: a weak shoulder peak near 1720 cm^−1^ (attributed to carbonyl C=O stretching), prominent bands at 1420 cm^−1^ (C-H bending, red area), 1150 cm^−1^ (C-O-C stretching in glycosidic linkages), and 910 cm^−1^ (α-1,6 glycosidic bond vibration, deep red area) [33]. Given that CD, AE, and pullulan are all polysaccharides, their spectral contributions are inherently overlapping, accounting for the high degree of similarity observed across all CD-AE-P film spectra in Figure 2a. Critically, no new peaks were detected, nor were any existing peaks abolished, across the tested CD concentration range, indicating the absence of covalent chemical modification and confirming physical compatibility among the components. Previous studies have indicated that the shifts in the O-H stretching vibration band might reflect the hydrogen-bonding interactions between components [27]. A slight blue shift of O-H stretching vibration peak was observed with increasing CD concentration, providing direct evidence for non-covalent hydrogen bonding interactions driving blend homogeneity.

#### 3.2.2. X-Ray Diffraction Analysis

XRD analysis was performed to probe the crystallinity of the constitute polymers and their blends. As shown in Figure 2b, the XRD patterns of AE-P bioactive films incorporating varying concentrations of CD exhibited broad diffraction responses with the maximum peak at approximately 20° (2θ), indicating the predominance of amorphous structures across all samples. It has been indicated that the diffraction peaks of CD were reported at 2θ = 6.5° (100), 10.5° (110), 12.5° (200), 15–20° (multiple peaks), and 27° and 35° [27,34]. In the present work, two distinct high-intensity diffraction peaks were observed at approximately 5.88° and 12.10°, assigned to the characteristic (100) and (200) lattice planes of CD associated crystalline phase in all CD-AE-P films. Critically, both peaks were shifted to higher 2θ angles with increasing CD concentrations (e.g., the (100), and the reflection shifted from 5.88° to 6.02° in CD20-AE-P), consistent with a progressive reduction in interplanar spacing (*d*), as calculated via Bragg’s law (n*λ* = 2*d*sinθ): *d*_100_ decreased from 0.38 nm to 0.37 nm, and *d*_200_ from 0.740 nm to 0.729 nm. This monotonic decrease in *d*-spacing was interpreted as quantitative evidence that elevated CD content promoted the formation of a more densely packed, nanoscale-ordered structure within the polymer matrix. Moreover, the diffraction intensities corresponding to the (100) and (200) reflection at 6.5° and 12.5° were significantly enhanced in CD15-AE-P and CD20-AE-P relative to formulations with lower CD content, suggesting preferential crystallographic orientation and improved crystallite alignment, which was likely attributed to the strengthened hydrogen-bonding interactions between the hydroxyl groups of CD and adjacent AE/pullulan chains, as corroborated by the blue shift of the O-H stretching band observed in Figure 2a.

#### 3.2.3. Small-Angle X-Ray Scattering Analysis

SAXS is primarily employed to analyze the non-periodic structures of amorphous and mesomorphic materials. The small-angle scattering intensity is associated with the electron density contrast between ordered and amorphous regions at nanoscale within the matrix [35]. Figure 2c,d presented the variation trends of scattering intensity in a small *q* region for AE-P bioactive films with varying CD concentrations. Within the range of 0.01 < *q* < 0.05 nm^−1^, the scattering intensities of all AE-P bioactive films exhibited similar profiles. Furthermore, an evident scattering peak was observed in CD0-AE-P at approximately 0.075 < *q* < 0.25 nm^−1^, indicating the formation of incomplete periodic structures with a thickness of ca. 55.58 nm (*d* = 2π/*q*). As CD concentration increased, the scattering peak was observed to shift progressively toward higher *q* values and to develop shoulder-like features. This peak was gradually attenuated in CD25-AE-P and CD30-AE-P. FTIR spectrum results has indicated that the addition of CD preferentially strengthened the hydrogen-bonding interactions within the matrix, likely promoting the rearrangement of macromolecules and disrupting the periodic structures in AE-P film. This effect was particularly pronounced in CD10-AE-P, CD15-AE-P, and CD20-AE-P, which exhibited correspondingly reduced *d*-spacing values. Moreover, these structural changes were corresponded by the slight increment in the diffraction angles corresponding to the (100) and (200) reflections of CD, as revealed by XRD analysis.

#### 3.2.4. Morphology Analysis

**Figure 3:**
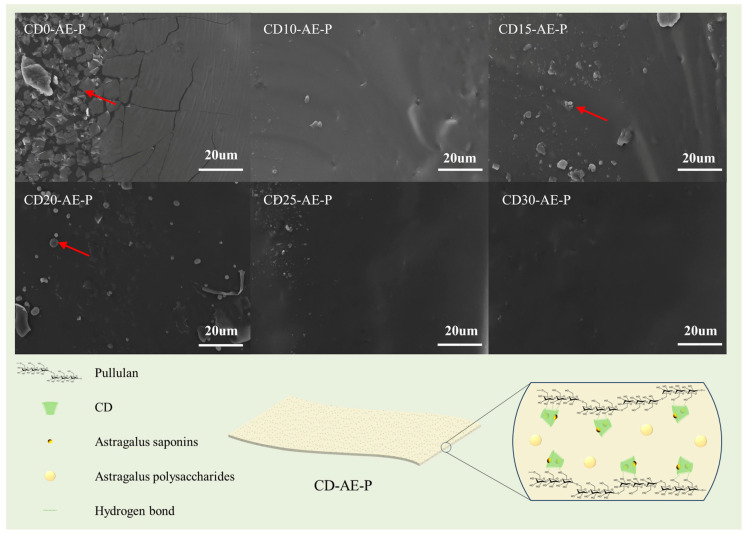
Fractured surfaces and structural schematic diagram of AE-P bioactive films with varying concentrations of CD.

The cross-section morphology of AE-P bioactive films incorporating varying concentrations of CD were analyzed via SEM images (Figure 3), which were naturally pre-fractured by liquid nitrogen. Notably, the CD0-AE-P sample exhibited a heterogeneous microstructure characterized by partial distinct fragments (indicated by red arrows), cracks, and structural discontinuities. In contrast, films incorporating CD displayed smoother and more continuous cross-sectional surfaces. Furthermore, as the content of CD increased, both the flatness and continuity of the corresponding films were improved, accompanied by some unevenness, small particles (red arrow), and protrusions in CD10-AE-P, CD15-AE-P, and CD20-AE-P samples. A previous study has indicated that, when pullulan was blended with pectin, the inter-chain voids within the inherently loose structure of pectin could be occupied by pullulan bundles, thereby yielding a more compact and morphologically continuous architecture [36]. Similarly, the intramolecular hydrogen bonding within pullulan was disrupted upon CD incorporation, particularly in CD25-AE-P and CD30-AE-P. The excessive cavity structures of CD enhanced the intermolecular interactions between pullulan and CD, leading to a random coiled rearrangement of pullulan chains and promoting the formation of more compact bundled aggregates. This structural evolution was corroborated by the SAXS results presented in Figure 2d.

### 3.3. Physico-Chemical Properties of CD-AE-P Films

#### 3.3.1. Thermal Properties (Tg)

**Figure 4:**
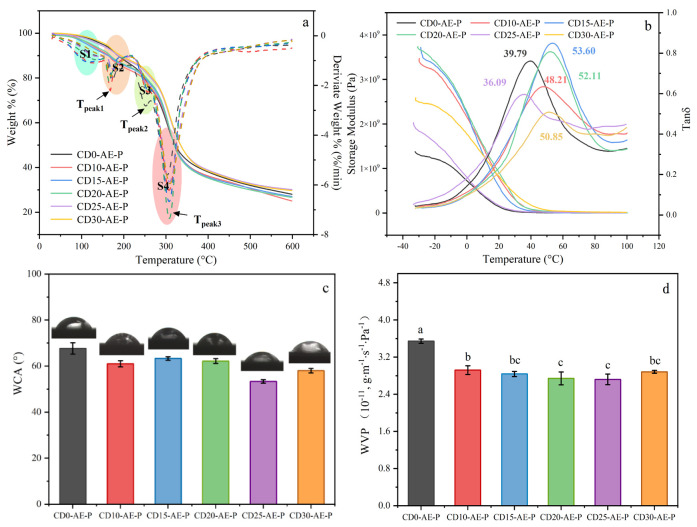
TG (**a**) and DMA (**b**) curves, WCA (**c**), and WVP (**d**) histograms of AE-P bioactive films with varying concentrations of CD. Lowercase letters within the same row in (**d**) indicate significant differences according to the ANOVA test (*p* < 0.05).

TGA is a fundamental technique widely employed to assess the thermal stability and degradation behavior of polymeric materials. It provides insights into compositional effects on thermal properties within polymer blends [30]. Figure 4a presents the TGA and DTG curves of AE-P bioactive films incorporating varying concentrations of CD. A comprehensive summary of the thermo-analytical data, including peak temperatures corresponding to the maximum weight loss rate at different stages, was provided in Table 1. All samples exhibited a four-step degradation profile: Stage 1 (S1), occurring below 150 °C, was attributed to the evaporation of free and physically absorbed water; Stage 2 (S2), spanning 150–220 °C, was primarily attributed to glycerol dehydration; Stage 3 (S3), between 220–270 °C, was assigned to glycerol decomposition; Stage 4 (S4), characterized by a sharp mass loss, was ascribed to the decomposition of functional groups and cleavage of the polymer backbone chains of CD, AE, and pullulan. As summarized in Table 1, the characteristic peak temperatures for S2 (T_peak2_) and S3 (T_peak3_) were systematically determined. Notably, an increase in T_peak2_ and T_peak3_ was observed upon CD incorporation, whereas further increases in CD concentration did not yield significant shifts, indicating that the hydrogen-bonding interactions between CD and glycerol were strengthened. Moreover, the hydroxyl-rich molecular architecture of CD facilitated enhanced intermolecular and intramolecular hydrogen bonding among CD, AE, and pullulan, thereby reinforcing the thermal stability of glycerol and improving the overall thermal robustness of the polymer blends.

#### 3.3.2. Dynamic Mechanical Analysis (DMA)

DMA was employed to investigate the temperature-dependent dynamic mechanical behavior of AE-P bioactive films incorporating varying concentrations of CD. These mechanical responses were intrinsically governed by the conformational mobility of polymer chains and the nature of intermolecular interactions, particulary hydrogen bonding and chain entanglement within the blend matrix [37]. DMA data were quantitatively reported as storage modulus (*E*′), loss modulus (*E*″), and damping factor (tan*δ* = *E*′/*E*″), which respectively reflected the elastic energy storage, viscous energy dissipation, and damping properties. Critically, DMA enabled precise identification of thermal transition, including glass transitions (T_g_), as well as secondary relaxation (e.g., *α*-relaxation and *β*-relaxation), thereby offering mechanistic insight into the compatibility, physicochemical, and rheological properties of the polymer components within blend system [38].

Figure 4b displayed the *E*′ and tan*δ* curves of AE-P bioactive films containing varying concentrations of CD. For all samples, the *E*′ values decreased monotonically with increasing temperature, a behavior directly attributed to thermally activated segmental mobility, which progressively reduced the chain rigidity and enhanced material compliance. Notably, a significant improvement in *E*′ was observed upon incorporation of CD, demonstrating effective reinforcement mediated by strong interfacial interactions, particularly hydrogen bonding between CD and pullulan matrix. Importantly, *E*′ exhibited a concentration-dependent maximum: it increased with CD loading up to 15–20 wt%, reaching peak values in CD15-AE-P and CD20-AE-P, and then declined at higher concentrations, indicating an optimal dispersion limit beyond which CD aggregation or localized plasticization might compromise reinforcing efficacy. The stiffening effect was mechanistically supported by two complementary structural changes induced by CD: (i) an increase in hydrogen-bonding density, as evidenced by attenuated O-H stretching band broadening in FTIR spectrum (Figure 2a), thereby restricting macromolecular chain mobility; (ii) enhanced local structural ordering, reflected by intensified X-ray scattering intensity at *q* = 0.11 nm^−1^, which promoted physical crosslinking and reinforced the elastic response. Collectively, these CD-induced structural modifications were found to synergistically improve the stiffness and elastic performance of CD-AE-P films [39].

The glass transition temperature (T_g_) of polymeric materials is operationally defined as the onset temperature of a rapid decline in mechanical resistance under a constant deformation rate, a signature of thermally activated, cooperative segmental motion that imparts enhanced chain mobility and flexibility. This transition is most sensitively detected as a distinct peak in the loss-tangent (tan*δ*) curve [40,41]. The *α*-relaxation, which coincided with the glass transition, reflected long-range, cooperative rearrangements of polymer chains that dominated energy dissipation in the viscoelastic regime [42]. As shown in Figure 4b, the tan*δ* profiles of AE-P bioactive films exhibited a single, well-defined relaxation peak across all CD concentrations, indicating the homogeneous phase behavior and good component compatibility within the amorphous matrix, and the observation was consistently corroborated by complementary structural evidence from SEM, confirming the absence of macroscopic phase separation. Notably, the value of T_g_ was systematically elevated upon the incorporation of CD relative to CD0-AE-P, with the tan*δ* peak shifting from 48.2 °C to a maximum of 53.60 °C in CD15-AE-P; concurrently, the tan*δ* peak was progressively broadened with increasing CD content up to 20 wt%, suggesting a widening distribution of relaxation times arising from heterogeneous local environments. The elevation in T_g_ was mechanistically attributed to two synergistic effects: (i) an increase in the density of intermolecular hydrogen bonds was facilitated by the additional hydroxyl groups introduced by CD, which restricted segmental mobility; and (ii) enhanced physical crosslinking via CD-mediated chain confinement, which reduced the effective free volume [43,44]. The pronounced effect observed in CD15-AE-P was attributed to optimal CD dispersion and maximal hydrogen bonding efficiency. In contrast, CD25-AE-P and CD30-AE-P exhibited a slight decrease in T_g_ (to 52.1 °C and 51.4 °C, respectively), yet they retained superior thermal stability, as evidenced by higher decomposition onset temperatures and delayed mass loss in TGA curves (Figure 4a), demonstrating that moderate CD overloading did not compromise the functional integrity. This robustness supported the suitability of CD-AE-P films for temperature-sensitive applications requiring both dimensional stability and controlled thermal response.

#### 3.3.3. Water Contact Angle (Wca) and Water Vapour Permeability (Wvp)

Water resistance is a critical performance criterion for food packaging materials. Figure 4c,d showed the water contact angle (WCA) and water vapour permeability (WVP) of AE-P bioactive films incorporating varying concentrations of CD. WCA is rigorously defined as the angle formed at the three-phase contact line, where the solid (film), liquid (water), and gas (air) phases intersect-between the tangent to the liquid–vapour interface and the solid surface, which serves as a key indicator of the hydrophilic or hydrophobic nature of the film surface; specifically, a lower contact angle corresponds to higher hydrophilicity [45].

Figure 4c shows the water contact angle (WCA) profiles for the AE-P bioactive film, incorporating varying CD concentrations. Following the widely adopted hydrophilicity/hydrophobicity classification criterion [46], a WCA > 65° was indicative of the hydrophobic surface character, whereas values < 65° were associated with hydrophilic behavior. CD0-AE-P exhibited a WCA of 66.7°. With increasing CD concentration, the value of WCA decreased progressively, reaching the minima of 53.0° and 58.0° in CD25-AE-P and CD30-AE-P, respectively. The systematic decline demonstrated that CD enrichment enhanced the surface wettability and hydrophilicity in a concentration-dependent manner, with the most pronounced effect observed at higher loadings. The underlying mechanism was attributed to two synergistic factors: (i) the introduction of abundant surface exposed hydroxyl groups from CD, which strengthened hydrogen bonding interactions with water molecules, as evidenced by sharpened and intensified O-H stretching bands in FTIR spectra (Figure 2a); (ii) CD induced surface topographical modification, resulting in nanoscale surface heterogeneity, including micro-particle dispersion and localized surface roughening, as visualized by SEM (Figure 3). According to wetting theory, such morphological features were found to reduce the surface energy and promote water spreading [47], consistent with the observed WCA decline. Furthermore, chemical composition (e.g., polar group density) and nanostructure (e.g., porosity, pore size distribution) are well-documented determinants of WCA [48]. Collectively, these results confirmed that CD functioned as a multifunctional modifier capable of rationally tuning the interfacial hydrophilicity of AE-P films.

WVP is a crucial quantitative indicator of a film’s moisture barrier performance. Packaging materials exhibiting low WVP values significantly suppressed moisture exchange between packaged food and the surrounding environment during storage, thereby retarding microbial proliferation, enzymatic activity, and product deterioration [49,50]. As presented in Figure 4d and Table 1, the WVP of AE-P bioactive films decreased progressively with increasing CD concentration. CD0-AE-P control exhibited a WVP of 3.54 (10^−11^, g·m^−1^·s^−1^·Pa^−1^), an incorporation of 25 wt% CD reduced the value to 2.72 (10^−11^, g·m^−1^·s^−1^·Pa^−1^), a 23.3% improvement in moisture barrier efficiency. This improvement was attributed to CD-mediated structural reinforcement: hydrogen-bonding interactions among CD, AE, and pullulan promoted the densification of molecular packing, thereby reducing intermolecular free volume and increasing the tortuosity of water diffusion pathways, as confirmed by SAXS analysis showing diminished scattering intensity and broadened correlation peaks. Notably, CD25-AE-P achieved the lowest WVP (2.72 × 10^−11^, g·m^−1^·s^−1^·Pa^−1^) and the lowest WCA (53.0°) among all formulations. This dual extremum was ascribed to CD’s intrinsic amphiphilic architecture: its hydrophilic exterior (rich in surface-exposed hydroxyl groups) enhanced interfacial hydration and surface wettability, whereas its hydrophobic internal cavities, occupied by astragaloside moieties, acted as selective nanoscale barriers that hindered bulk-phase water transport. Consequently, CD simultaneously optimized both surface–water affinity and matrix-level diffusion resistance, yielding a synergistic improvement in comprehensive moisture management.

### 3.4. Antioxidant Capacity, Migration Behavior, and Anti-Browning of Sliced Apples

**Figure 5:**
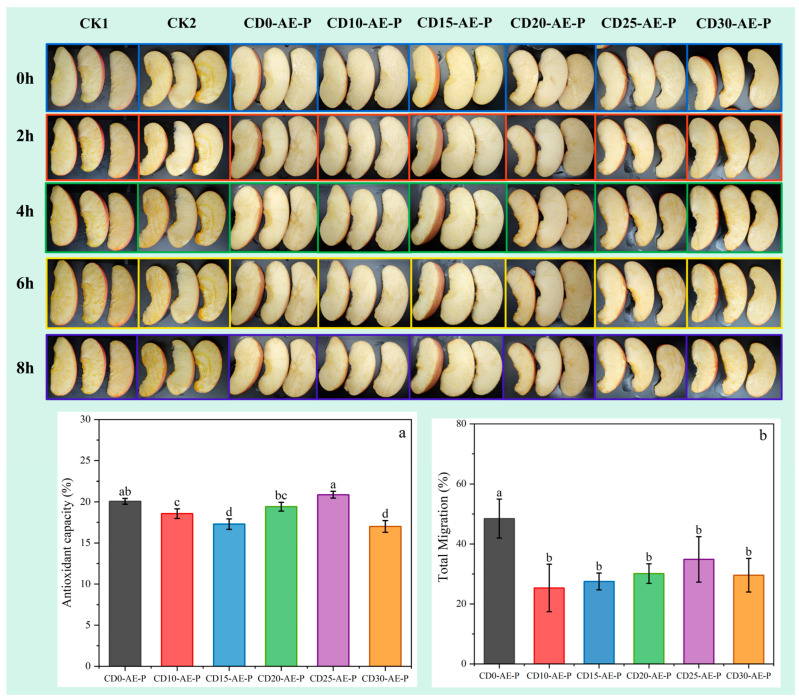
Morphological changes of fresh-cut apples slices immersed with water (CK1), pullulan (CK2), and CD-AE-P film-forming solutions stored at 25 °C; Antioxidant activity of CD-AE-P bioactive films (**a**); Total migration (%) of CD-AE-P bioactive films immersed in distilled water (**b**). Lowercase letters within the same row indicate significant differences according to the ANOVA test (*p* < 0.05).

Figure 5a,b presented the antioxidant capacity (%) and total migration behavior of CD-AE-P bioactive films. As shown in Figure 5a, the antioxidant capacity exhibited a modest yet consistent decline with increasing CD concentration. In Figure 5b, it was evident that the total migration value of AE-P bioactive films significantly decreased with the addition of CD, while no significant differences were observed among the films containing varying concentrations of CD. Notably, both characteristics displayed lower values in CD10-AE-P, CD15-AE-P, and CD20-AE-P film samples.

Furthermore, Figure 5 displayed photographic evidence of fresh-cut apple slices treated with distilled water (CK1), pullulan solution (CK2), and CD-AE-P film-forming solutions and stored at 25 °C for 8 h to accelerate oxidative browning. Both control groups (CK1, CK2) exhibited rapid, extensive surface browning within 4 h, as indicated by red arrows, accompanied by visible tissue shrinkage and surface dehydration. In contrast, CD0-AE-P and CD25-AE-P groups showed only scattered, localized brown spots after 4 h. All CD-AE-P-treated samples maintained vibrant, visually appealing coloration throughout the storage period, with CD10-AE-P, CD15-AE-P, and CD20-AE-P groups demonstrating the strongest protective effect.

The browning discoloration observed on the cut surface of apples is a complex enzymatic process predominantly mediated by the oxidation of phenolic compound, catalyzed primarily by polyphenol oxidase (PPO) and peroxidase (POD), and the oxidative cascade ultimately led to the formation of brown polymers [15]. Consequently, the release of AE was found to be directly associated with suppression of PPO and POD activities, thereby impeding enzymatic browning. However, the release of AE was modulated by the antioxidant capacity and migration behavior of AE from CD-AE-P bioactive films. The latter was governed by a multifaceted interplay of physicochemical mechanisms, including diffusion, degradation, dissolution, and interfacial mass transfer [51]. Under simulated liquid food-contact conditions (e.g., immersion in distilled water), water immigration was facilitated by the hydroxyl groups presented in hydrophilic CD-AE-P films. This hydration-induced plasticization triggered macromolecular swelling and pore expansion within the film matrix, thereby enhancing both the dissolution and migration rates of AE. As shown in Figure 5, the reduced antioxidant capacity values, lower total migration rates, and lighter colors intensities indicated that CD incorporation significantly retarded the migration of AE. This effect was most pronounced in CD10-AE-P, CD15-AE-P, and CD20-AE-P groups. Furthermore, the inherent hydrophilicity of distilled water and surfaces of sliced apples promoted sustained release of AE. Collectively, these findings confirmed that AE was successfully encapsulated and stabilized via host–guest inclusion complexation with CD, enabling effective delay of surface browning and extension of shelf life in fresh-cut apples.

### 3.5. Volatile fingerprints analyzed by GC-IMS

**Figure 6:**
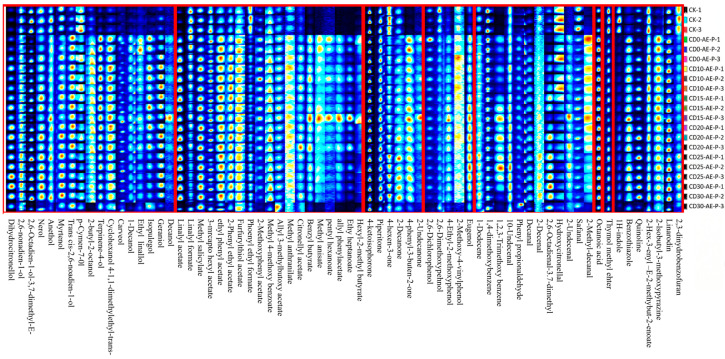
Fingerprints of VOCs in pre-treated fresh-cut apples slices with storage.

The GC-IMS technique has been successfully employed for the separation of isomers and isobaric volatile compounds, showing the advantages of high sensitivity, minimal sample/environmental requirements, and intuitive, three-dimensional data visualization [52]. As shown in Figure 6, fingerprint profiles derived from 3D topographical plots were utilized to identify and perform semi-quantitative assessment of volatile organic component (VOC) accumulations in apple slices preprocessed and subsequently stored at 4 °C for 48 h. VOC signal intensities were encoded chromatically: blue represented the background signal, whereas yellow and red denoted progressively higher relative abundances. Each vertical column in Figure 6 corresponded to a distinct volatile compound; a total of 68 resolved signals were identified in the processed apple slices, comprising 17 alcohols, 19 esters, 6 ketones, 5 phenols, 3 alkenes, 9 aldehydes, 1 acid, and 1 ether.

**Figure 7:**
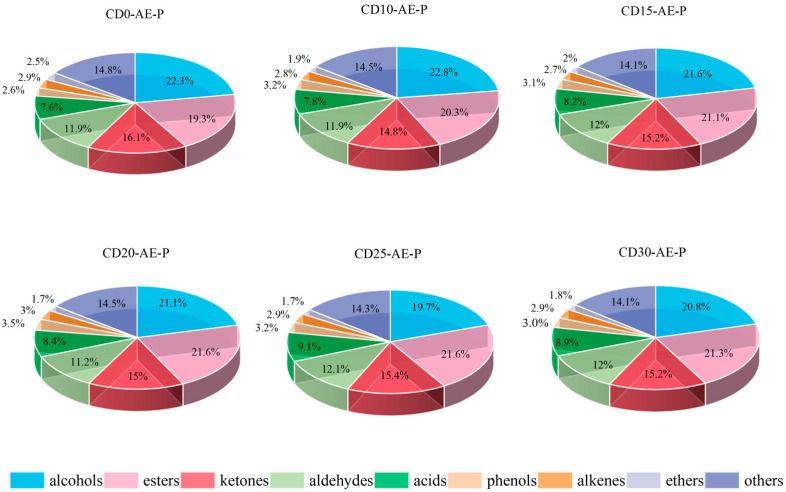
Quantitative comparison of VOCs in fresh-cut apples slices with storage obtained by GC-IMS.

Studies have shown that esters, aldehydes, and alcohols constituted the predominant volatile organic compounds (VOCs) in fresh-cut apples. Among these, esters were primarily responsible for fruity and floral aroma notes, whereas aldehydes and alcohols contributed significantly to the characteristic fresh aroma [53]. During the storage, three interrelated biochemical pathways could be occurred in fresh-cut apples: 1. volatile esters, the major contributors to fruity aroma, underwent progressive loss via volatilization or enzymatic reactions, such as unsaturated fatty acids, and were oxidized under catalysis of lipoxygenase, generating secondary aldehydes and alcohols with off-odor characteristics. 2. Exposure of phenolic compounds to oxygen in the presence of polyphenol oxidase (PPO) could catalyze their oxidation to quinones, triggering enzymatic browning on the surface and reducing phenolic substances. 3. The proliferation of microorganisms and anaerobic respiration promoted the formation of ketone substances, which were associated with musty or unpleasant aroma [54,55]. As shown in Figure 7, alcohol, esters, and ketones collectively accounted for approximately 60% of the volatile compounds in all sliced apple sample groups. Specifically, in the CK group, alcohol, esters, and ketones constituted 19.6%, 18.1%, and 18.4% of the total volatiles, respectively. In contrast, sliced-apple samples immersed in the CD-AE-P solution exhibited elevated levels of alcohols (19.7–22.8%) and esters (19.3–21.6%), indicating a concentration-dependent increase relative to the CK group. In contrast, ketone accumulation in all CD-AE-P groups (14.8–16.1%) decreased significantly relative to CK group (18.4%). Concurrently, phenol content in all sliced-apple samples treated with CD-AE-P solutions averaged approximately 3.0%, compared to 2.5% in the CK group. These results indicated that CD-AE-P treatment effectively limited oxygen exposures, thereby suppressing the oxidation of polyphenol and subsequent quinones formation, evidenced by higher residual polyphenol levels and reduced surface browning in CD-AE-P groups (Figure 5). Furthermore, the volatilization of esters and lipoxygenase-catalyzed lipid oxidation were both inhibited by the immersion treatment, contributing to better retention of fresh fruity aroma and mitigating excessive ketone accumulation. However, the anaerobic respiration was inevitably triggered by the oxygen barrier effect, leading to elevated alcohol levels in CD-AE-P groups. Notably, the accumulation of alcohols and esters showed a downward trend. However, they showed an upward trend as the amount of β-cyclodextrins (CD) was increased, accompanied by the minimum and maximum values in the CD25-AE-P group, which indicated that the treatment by CD25-AE-P was the optimal formulation for preserving the sensory and chemical freshness of fresh-cut apples.

## 4. Conclusions

The impact of β-cyclodextrins (CD) concentration on the multi-scale structural organization and physicochemical properties of pullulan/*Astragalus* extract (AE) bioactive films (CD-AE-P), and its subsequent influence on the migration behavior, anti-browning, and volatile compounds modulation in fresh-cut apple slices, has been systematically investigated. Incorporation of CD enhanced the intermolecular H-bonding interactions among hydroxyl-rich components, inducing macromolecules rearrangement, disruption of long-range periodic structures, and formation of relative ordered configurations characterized by reduced interplanar spacings. Furthermore, the rearrangement of pullulan chains and disputed structures promoted the formation of compact bundled structures, evidenced in CD25-AE-P and CD30-AE-P. The reinforced H-bonding network significantly restricted segmental mobility of polymer chains, thereby improving both the thermal stability of glycerol and the storage modulus of CD-AE-P films; notably, the highest T_g_ value was observed in CD15-AE-P, suggesting an optimal balance between crosslinking density and chain flexibility at the CD loading. Furthermore, while CD introduction marginally increased overall hydrophilicity of the films due to its abundant surface hydroxyl groups, its toroidal cavity structure sterically hindered deep water penetration, resulting in a net reduction in water vapor permeability (WVP). Critically, the intensified intermolecular interactions, including H-bonding, and steric constraints impeded the diffusion of AE, which prolonged the antioxidant efficacy of AE during storage. In contrast, the hydrophilic nature of both the film surface and the apple tissue interface facilitated AE release with hydrated conditions, contributing directly to effective suppression of browning and favorable retention of aroma-active volatiles in fresh-cut sliced apples. Collectively, these results demonstrate that rational design of CD-modified pullulan films, through precise control of CD content, enabled synergistic tuning of structural hierarchy, barrier functionality, and active compound delivery, offering a promising strategy for next-generation bioactive packaging in the fresh-cut produce industry.

## Figures and Tables

**Table 1:** Peak temperatures, WVP, and WCA values of AE-P bioactive films with varying concentrations of CD. Lowercase letters within the same row indicate significant differences according to the ANOVA test (*p* < 0.05).

	CD0-AE-P	CD10-AE-P	CD15-AE-P	CD20-AE-P	CD25-AE-P	CD30-AE-P
T_peak1_ (°C)	172.69	167.70	171.49	165.07	176.44	170.35
T_peak2_ (°C)	251.90	256.91	257.18	259.45	258.77	261.87
T_peak3_ (°C)	302.98	307.07	307.99	308.16	308.78	309.45
WCA (°)	66.7 ± 2.5 ^a^	61.0 ± 1.3 ^b^	63.3 ± 0.8 ^b^	62.2 ± 1.0 ^b^	53.3 ± 0.8 ^c^	58.0 ± 1.0 ^d^
WVP (10^−11^, g·m^−1^·s^−1^·Pa^−1^)	3.54 ±0.05 ^a^	2.92 ± 0.09 ^b^	2.84 ± 0.06 ^bc^	2.74 ± 0.14 ^c^	2.72 ± 0.12 ^c^	2.88 ± 0.03 ^bc^

## Data Availability

The original contributions presented in this study are included in the article. Further inquiries can be directed to the corresponding authors.
